# The Interplay Between Body Weight and the Onset of Puberty

**DOI:** 10.3390/children12060679

**Published:** 2025-05-25

**Authors:** Alexandros K. Kythreotis, Marina Nicolaou, Eirini Mitsinga, Habib Daher, Nicos Skordis

**Affiliations:** 1 Derby Hospital, Derby DE22 3NE, UK; kyprianos.kythreotis1@nhs.net; 2 Addenbrookes Hospital, Cambridge CB2 0QQ, UK; 3 Barts and The London School of Medicine and Dentistry, Queen Mary University of London, London E1 2AD, UK; e.mitsinga@smd20.qmul.ac.uk; 4 School of Medicine, University of Nicosia, 2414 Nicosia, Cyprus; 5 Division of Paediatric Endocrinology, Paedi Center for Specialized Paediatrics, 2025 Nicosia, Cyprus

**Keywords:** puberty, obesity, endocrine disruptors, insulin, adipokines

## Abstract

This overview explores the complex relationship between environmental factors, particularly obesity, and the timing of puberty, with a focus on how hormonal and genetic interactions are influenced by external conditions. Puberty (gonadarche) is characterised by the activation of the hypothalamic–pituitary–gonadal (HPG) axis. The onset and progression of puberty vary significantly among individuals, primarily due to genetic factors, with key genes like kisspeptin 1 (*KISS1*) and makorin ring finger protein 3 (*MKRN3*) playing a crucial role. Cohesively, this paper emphasises that environmental factors, particularly obesity and exposure to endocrine-disrupting chemicals (EDCs), have become significant influences on the timing of puberty. Childhood obesity has risen significantly in recent decades and the age of pubertal onset has declined over the same period. Obesity greatly disrupts hormone regulation in pre-pubertal children. Leptin accelerates the onset of puberty in girls but not in boys. The underlying mechanism is proposed to be the increase in *Kiss1*/*GnRH* signalling. On the contrary, excess leptin in boys suppresses testosterone production by increasing oestrogen conversion. Low adiponectin in obese girls may contribute to earlier puberty due to a reduced inhibition of *Kiss1*/*GnRH* signalling. Low adiponectin in boys is linked to delayed puberty due to its role in maintaining insulin sensitivity and testosterone production. Hyperinsulinemia influences pubertal timing through central and peripheral mechanisms. Insulin acting synergistically with leptin promotes the earlier onset of puberty in girls but not in boys. The effects of exposure to certain EDCs—mostly obesogenic chemicals that mimic the action of natural hormones—on the timing of puberty remain unclear; hence, further research on this topic is needed. Addressing and preventing obesity in children could potentially mitigate these alterations in pubertal timing.

## 1. Introduction

Puberty is defined as the physiological process by which a child achieves physical and sexual maturity and reproductive ability [1]. During puberty, the release of pubertal hormones induces a wide array of changes, encompassing emotional, biological, and physical transformations [1,2]. The release of sex steroids is governed by the hypothalamic–pituitary–gonadal (HPG) axis, which is activated after the age of 8 years and 9 years in girls and boys, respectively; collectively, these changes are known as gonadarche. Prior to gonadarche the reticularis zone within the adrenals is often activated, resulting in adrenarche. The activation of the HPG axis is determined by genetic loading and influenced by environmental factors. Certain genes need to be activated or inhibited, which leads to the release of certain hormones. The genetic variations among individuals largely account for the significant variability observed in pubertal development [3,4].

The exact timing of puberty remains a mystery and, as such, various studies have identified the effects that intrinsic genetic pathways have on the onset of puberty. Considering the fact that puberty is also affected by external sources such as environmental factors and nutrition, this paper focuses on the effects that the environment has on pubertal timing [5]. Over the last century, the average age that children enter pubertal development puberty has dropped by 3 years and, approximately every 25 years, this age drops by around 6–9 months. This statistic follows secular trends that have been observed in recent changes regarding average childhood body mass index (BMI) figures, showing that, compared with 20 years ago, more children nowadays are either on or above the 95th percentile of the BMI, clinically classifying them as obese [6,7]. In addition to an increasing BMI, there has also been an increasing trend in the consumption of EDCs among young children. Moreover, obesity causes disturbances to the release of pubertal hormones by disrupting both central and peripheral signalling pathways. This ranges from simple disruptions in the HPG axis to complex changes to genetic components that regulate the endocrine milieu. This review aims to examine how obesity and environmental factors exert an effect on the HPG axis and how that in turn alters the timing of sexual maturation.

## 2. The Timing of Puberty

The onset of puberty is primarily driven by gonadarche, a critical process involving the reactivation of the HPG axis, which subsequently leads to the production and release of gonadal hormones [5,8]. This activation of the axis is followed by the hypothalamus starting to release GnRH in a pulsatile manner, which is subsequently detected by the pituitary, prompting it to trigger a hormonal response. This response starts releasing follicle stimulating hormone (FSH) and luteinising hormone (LH), which in turn triggers a response in the gonads [9,10]. However, the knock-on effects this has differs between boys and girls. In boys, FSH causes increased sperm production and LH causes an influx of testosterone. In girls, FSH stimulates an increase in oestrogen, while LH promotes a rise in progesterone [11]. These gonadal hormones in turn exert a negative feedback effect on the hypothalamus and pituitary, inhibiting the release of *GnRH* and gonadotrophins, respectively [12,13]. This collectively regulates the axis via the aforementioned loops, which regulate hormonal production during puberty and thus control the stages it goes through [14]. It is imperative to keep in mind that these endocrinological pathways are affected by a myriad of genetic, endocrinological, and, most importantly, environmental factors [1]. Factors such as ethnicity play a detrimental role in the timing of puberty, as studies have suggested. For instance, African-American and Asian children have a tendency to initiate puberty earlier than Caucasian, Hispanic, and European children [15,16].

### 2.1. Genetic Factors

The onset of puberty varies greatly amongst individuals. This is mainly because it is affected by a multitude factors, one of them being genetic. For this reason, researchers often consider an age range for the onset of puberty rather than a set age. The range is set as 8–13 years and 9–14 years for girls and boys, respectively [1].

Certain genes must be activated to initiate essential hormone release vital to the initiation of puberty. The genes involved are categorised as follows: puberty-activating (PA), puberty-inhibiting (PI), and dual-effect genes. The main PA genes are *KISS1*, *Neuropeptide Y* (*NPY*), and *Leptin* (*LEP*) as they play a role in the onset of puberty and stimulation of the HPG axis [3,17].

Cohesively, the main PI genes are *Gamma*-*aminobutyric acid* (*GABA*), *Delta*-*like Non*-*canonical Notch Ligand 1* (*DLK1*) and *MKRN3*, which are all expressed in the hypothalamus and prevent puberty from starting until the child is capable of coping with the physiological changes. Increasing the level of expression of those genes delays puberty. Conversely, when there are mutations that reduce their expression, puberty is initiated at an earlier age [18].

The KISS1 gene, which is expressed in the hypothalamus, signals the release of GnRH from itself. This happens post KISS1 binding to its receptor, KISS1R [19,20]. This gene is inhibited by the DLK1 gene, subsequently halting early-onset puberty [19]. This can only be accomplished when genes MKRN3, DLK1, and GABA are inhibited, which allows for the KISS1 gene to bind to its receptor [21,22,23].

### 2.2. Adrenarche

Adrenarche refers to the process of maturation of the adrenal glands and the increased production of dehydroepiandrosterone (DHEA) and dehydroepiandrosterone sulphate (DHEAS) [24,25]. The exact underlying mechanism for the activation of the zona reticularis of the adrenals is still unknown but it could be influenced by the hypothalamic–pituitary–adrenal (HPA) axis. Androgens in turn activate the HPG axis, implying that through an indirect pathway, the HPA axis plays a role in the onset of puberty [26]. Oestradiol enhances the activity of the HPA axis, facilitating the activation of the HPG axis, whereas testosterone suppresses the activity of the HPA axis, thus indirectly influencing the HPG axis [27].

## 3. Body Weight and Puberty

### 3.1. Childhood Obesity

Obesity is among the most prevalent chronic conditions in children and adolescents. It is typically defined using the BMI, considering the individual’s weight, height, and age. According to the World Health Organization (WHO), for children aged 5–19, obesity is determined by a BMI-for-age that is more than two standard deviations above the WHO Growth Charts. For children less than 5 years of age, obesity is defined as a weight-for-height greater than three standard deviations above the WHO Child Growth Standards median [28]. According to the United Nations Children’s Fund (UNICEF), the number of children under the age of 5 years that were overweight or obese increased from 30 million in the year 2000 to 37 million in 2024. The NCD Risk Factor Collaboration (NCD-RisC) further supports this increasing trend as it estimated that the percentage of overweight and obese children aged 5 to 19 years increased from 4% in 1975 to approximately 20% in 2022 [29]. The underlying causes behind these obesity trends are complex and involve multiple factors, often due to intricate interactions between biological, genetic, socioeconomic, environmental, and cultural influences [30].

### 3.2. EDCs and Puberty

In recent years, particular attention has been drawn towards environmental factors such as EDCs, which are known for their ability to imitate natural hormones and disrupt the hormonal balance, contributing to the modern obesity epidemic [31]. Moreover, the rising prevalence of EDCs, coupled with their potential to interfere with the endocrine system, has sparked interest among researchers investigating the link between EDCs and the altered onset of puberty observed in recent times.

As mentioned, EDCs have the ability to interfere with the hormonal regulation of puberty through various mechanisms. For example, they may cause epigenetic changes in pubertal genes; act on peripheral tissues like fat cells or adrenal glands, increasing adrenal androgen levels; or influence pathways in the central control systems of puberty through the HPG axis [32]. In addition, some EDCs have oestrogenic activity and can mimic naturally occurring oestrogens in the body [33,34]. A recent study showed that early pre-pubertal exposure to even very low, environmentally relevant doses of common EDCs, such as Bisphenol A (BPA), could accelerate the onset of puberty yet decrease reproductive parameters in female mice [35]. Similarly, another EDC that has been studied for its ability to influence the reproductive system is the plasticiser bis (2-ethylhexyl) phthalate (DEHP). A study exposing juvenile rats to DEHP levels comparable to exposure levels in Italian children during particularly vulnerable peri-pubertal periods revealed sex-specific metabolic disruptions. In boys, DEHP exposure delayed reproductive development, suggesting anti-androgenic effects, while in girls, the thyroid emerged as a potential target for DEHP toxicity [36,37]. Furthermore, exposing female rats to a mixture of oestrogenic and anti-androgenic EDCs revealed that although the first generation remained largely unaffected, subsequent offspring generations (F2 and F3) experienced significant reproductive issues. These issues included decreased GnRH interpulse intervals, delayed vaginal opening, and irregular reproductive cycles [37,38]. Although associations between EDCs and altered pubertal timing have been observed, the current literature is limited and inconsistent, preventing conclusive evidence of a causal relationship. Analyses are complicated by the long latency period between EDC exposure and pubertal outcomes coupled with mixed exposure throughout life and the challenge of distinguishing EDC effects from those of nutrition and adiposity [39]. Additionally, identifying the critical windows of time during which exposure to EDCs has more pronounced effects is challenging. This is largely due to the persistent presence of EDCs throughout daily life. The current literature discussing pre- and post-natal exposure to EDCs has proved to be inconclusive. However, it has been suggested that post-natal exposure to phthalates is associated with pubertal disruptions in girls, such as thelarche and later pubarche [39]. Further prospective and longitudinal studies are needed to clarify the effects of EDCs on puberty and reproductive health, determine critical exposure periods, and elucidate the underlying mechanisms involved.

## 4. Adipokines (Leptin and Adiponectin)

The hormones secreted from adipose tissue have a sexually dimorphic impact on the HPG axis. Adipokines, particularly leptin and adiponectin, have been found to interact with the HPG axis by crossing the blood–brain barrier (BBB) [40]. They are both secreted from adipose tissue and play a crucial role in maintaining energy balance. Additionally, they have different mechanisms by which they exert effects on the HPG axis in opposing aspects. In obesity, there is an increased amount of adipose tissue because there is an increased level of adipokines [41]. There is a positive association between the early onset of puberty and increased body weight, mostly in girls [42,43].

Leptin regulates reproductive functions by stimulating the release of gonadotrophins via directly influencing pubertal gene expression [44]. In girls, elevated leptin levels signal excess energy storage, which is crucial for supporting the growth and development associated with puberty [45]. Typically, healthy girls see a rise in leptin before GnRH pulsatile secretions, which may explain why it causes early puberty in girls. Moreover, leptin also affects the genes associated with initiating puberty. Studies have shown that leptin receptors (LEPR) on the hypothalamus influence KISS1 expression. More specifically, a surplus of leptin is associated with an increased expression of KISS1 [46]. Obesity enhances leptin resistance that precipitates limitations in the effects of leptin; therefore, a far greater amount of leptin is required to induce the feeling of satiety [47,48]. This can be observed in obese girls, where excess leptin may lead to early KISS1 activation, causing precocious puberty. In leptin-deficient states (e.g., malnutrition and extreme leanness), KISS1 expression is suppressed, delaying puberty.

Furthermore, leptin has been found to affect LH more profoundly than other hormones as individuals with higher levels of leptin are also associated with having greater levels of LH. This further supports a strong correlation between leptin and LH [49]. Higher levels of LH cause an increased production of oestrogen, which also results in an increased release of leptin, feeding a cyclical trend [47]. This process is initiated when oestrogen stimulates the expression of the Ob gene in adipose tissue, triggering an increase in the production and secretion of leptin [50]. Leptin also interacts with both glutamate and *GABA* genes, albeit to a lesser extent in girls. Moreover, leptin enhances the expression of steroidogenic genes, including cytochrome P450 19A1 (CYP19A1), thereby promoting oestrogen biosynthesis in girls [47]. In conclusion, leptin exerts multiple effects on pubertal girls, primarily by triggering or allowing an earlier-than-normal onset of puberty.

**Figure 1:**
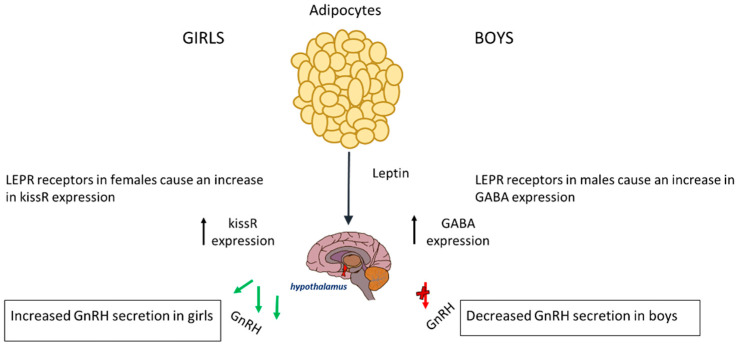
The release of leptin from adipocyte tissue has different effects on each gender. In boys, it causes increased expression of GABA and thus a decreased expression of GnRH. In girls, it causes an increase in KISSR expression.

In boys, the relationship between leptin and puberty is more complex and less clearly understood; however, boys with obesity enter puberty at a later age [46]. Usually, in boys of normal weight, leptin is reduced before the onset of puberty, suggesting that a small drop may be required for pubertal initiation. The excess leptin seen in obese boys affects pubertal onset via genetic pathways and alterations in the hypothalamus [51]. In boys, the LEPR that leptin binds onto in the hypothalamus has a substantially different effect on the HPG axis; it causes an increased expression of *GABA*, leading to a delay in the onset of puberty. This is because a rise in *GABA* prevents pulsatile releases of GnRH, which is needed for the initiation of puberty [52]. Therefore, boys suffering from obesity also have elevated levels of *GABA* neurotransmitters, which can subsequently delay puberty. The rise in *GABA* causes a reduced expression of PA genes in mechanisms that are not fully understood [53]. Leptin increases aromatase activity, leading to a higher oestrogen-to-testosterone ratio that may explain the delayed puberty seen in obese boys [54,55]. The effect of leptin on the timing of puberty is represented in Figure 1.

Another adipokine of interest, adiponectin, also plays a role in the onset of puberty. When adiponectin binds to its receptors *Adiponectin Receptor 1* (*AdipoR1*) and *Adiponectin Receptor 2* (*AdipoR2*), which are found in the hypothalamus, it exerts an inhibitory effect on the release of kisspeptin, thereby delaying the progression of puberty. This in turn inhibits the release of *GnRH*, leading to a delay in the onset of puberty. Studies have shown that individuals with central obesity experience lower levels of adiponectin via mechanisms that are not fully understood [56]. This might be a result of increased adiponectin resistance due to a reduced expression of the *AdipoR2* receptor [57]. This reduction is secondary to the dysfunction in adipose tissue caused by increased insulin resistance, increased secretion of inflammatory cytokines, and chronic low-grade inflammation. These factors contribute to a high leptin-to-adiponectin (L/A) ratio in the systemic circulation. A high L/A ratio disrupts the initiation of puberty differently for boys and girls. Adiponectin levels decline significantly upon the onset of puberty, with boys experiencing a more pronounced drop than girls [58]. Thus, when obesity coincides with puberty, it further alters adiponectin levels, which in turn affects pubertal timing.

Obesity is associated with a low-grade chronic inflammatory state, characterised by the elevation of pro-inflammatory cytokines, including but not limited to interleukin-6 (IL-6) and tumor necrosis factor-alpha (TNFα). This inflammatory state reduces the inhibitory regulation of pubertal initiation, potentially contributing to an earlier onset of puberty in girls [54]. Furthermore, when girls begin puberty with an increased BMI, their adiponectin levels are reduced even further. Adiponectin affects puberty by interacting with PA genes, causing a reduction in the bioavailability of kisspeptin to bind to the GPR54 receptors in the hypothalamus. It should be noted that although its levels are inversely correlated with circulating LH levels, the mechanisms behind this are yet to be fully understood [59,60]. However, based on its interactions with the HPG axis, it can be concluded that adiponectin ultimately contributes to disruption in the onset of puberty, albeit to a lesser extent. Furthermore, due to the lack of current literature on the matter, more research is needed to understand the specific mechanisms in which adiponectin affects pre-pubertal girls and contributes to earlier-onset puberty.

Similarly, adiponectin affects boys in comparable ways. Physiologically, its levels drop prior to puberty in boys, a decline that is both more rapid and extensive than in girls. Therefore, in boys, there is increased inhibition of the HPG axis by adiponectin, causing delayed puberty. Adiponectin is inversely associated with androgen levels because it increases the levels of sex hormone-binding globulin (SHBG), thereby decreasing free testosterone [61]. In contrast to girls, the reduction in adiponectin in boys causes delays in puberty as adiponectin plays a crucial role in maintaining insulin sensitivity and testosterone production. These correlations help us to understand the mechanisms by which adiponectin influences puberty in obese boys. To elaborate, the rising levels of androgens starting from adrenarche cause a greater decrease in adiponectin in boys from a younger age than in girls [59]. Adiponectin is also involved in the suppression of the secretion of both LH and FSH, influencing gonadal activation. The mechanisms described above result in delayed testicular maturation, culminating in a delay in the onset of puberty. This statistic alone allows for the suggestion that obesity causes earlier-onset puberty in boys, presenting a conflicting dilemma.

The studies that were used to identify the delay in initiation of puberty were cross-sectional epidemiological studies that focused on Asia [59]. Other cross-sectional epidemiological studies undertaken in the United States and Europe have shown that obese boys tend to have earlier-onset puberties [46]. Puberty is a multifactorial process and, although many studies show that obesity delays puberty in boys, the overall evidence remains inconclusive. The need for further literature and research is crucial to truly grasp the effects that adiponectin and obesity have on pubertal development in boys.

## 5. Insulin

Puberty is naturally characterised by a state of physiological insulin resistance and a reduction in insulin sensitivity. This occurs even in average-weight, non-obese girls and boys, and is associated with changes in circulating insulin levels [62]. However, obesity exacerbates insulin resistance and this has a sexually dimorphic impact on the timing of puberty [63].

**Figure 2:**
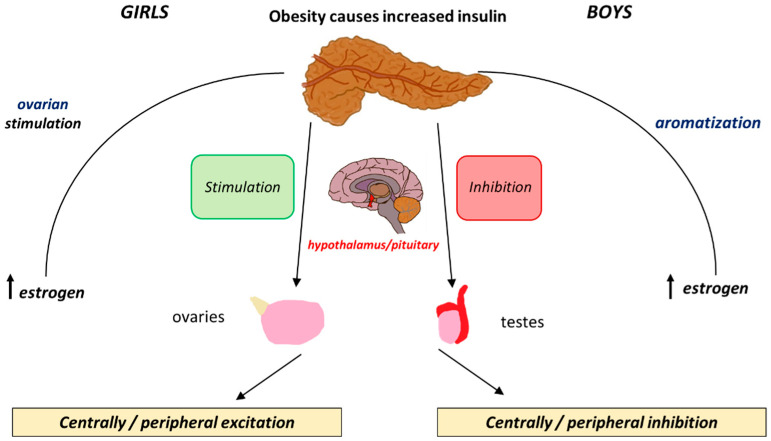
The differential effects that insulin exerts peripherally and centrally and how these cause positive and negative feedback loops in boys and girls.

The effects of insulin levels on the HPG axis are similar to those of leptin. Both hormones are involved in energy homeostasis and metabolism and their interplay becomes especially relevant during puberty. Insulin promotes leptin production in adipose tissue and also acts directly on the HPG axis. It exerts its influence by binding to insulin receptors (IRs) in the hypothalamus and pituitary, thereby stimulating *KISS1* gene expression and promoting the pulsatile release of *GnRH* [64]. In addition, insulin enhances the production of sex steroids, especially oestrogens, which are crucial for pubertal development [65]. In obese girls, elevated oestrogen levels synthesised in adipose tissue further contribute to the early onset of puberty. The positive feedback mechanism by which insulin affects both the central and peripheral components of puberty is illustrated in Figure 2.

The relationship between insulin and puberty in boys is more complex. Obese boys may experience delayed puberty, potentially due to the suppression of the HPG axis by insulin resistance. Obese boys have low levels of free testosterone and increased levels of oestrogens due to increased aromatisation that acts as an inhibitory mechanism for gonadotropin release. Although there is no clear delay, obese boys tend to show a delay in pubertal onset [66,67,68] (Table 1).

## 6. SGA, Obesity, and Premature Gonadarche

Small for gestational age (SGA) is defined as a birth weight at or below the 3rd percentile or less than two standard deviations below the mean for gestational age [69]. SGA is often a consequence of inadequate nutrition during foetal development. Over recent decades, studies have demonstrated a strong association between a low birth weight and childhood obesity. SGA infants are at increased risk of long-term health complications, including disrupted growth patterns, compared with infants with a normal birth weight. One of the most well-documented long-term outcomes is the development of obesity, often emerging during childhood and potentially affecting the timing of pubertal onset [70]. This increased risk can be attributed to compensatory growth, where SGA children undergo rapid post-natal growth in an attempt to reduce developmental disparities with their peers. However, this compensatory growth may overshoot, leading to accelerated weight gain and, ultimately, obesity [71].

The exact biological mechanism linking SGA to obesity is incompletely understood [72]. It is hypothesised that impaired foetal development in SGA newborns alters insulin sensitivity, thereby increasing the risk of metabolic dysfunction and childhood obesity [73]. This disruption of development is also believed to affect the timing of puberty, often resulting in an earlier pubertal onset in both boys and girls. In particular, SGA babies, especially girls, frequently experience early adrenarche, marked by elevated circulating adrenal androgens, which may prematurely stimulate the HPG axis [73]. Both sexes appear to exhibit a similar pattern, with SGA and subsequent obesity acting synergistically to trigger earlier puberty. Given these findings, strategies aimed at monitoring growth trajectories and preventing excess weight gain in SGA infants are crucial for mitigating adverse pubertal and metabolic outcomes.

## 7. Conclusions

This review highlights the complex relationship between childhood obesity and the timing of pubertal onset, with a focus on endocrine changes involving adipokines and insulin that mediate these effects in a sex-specific manner. Adiponectin plays a modulatory role in puberty through its regulation of *KISS1*/*GnRH* signalling and metabolic pathways. A natural decline in adiponectin is a normal part of puberty but in obesity, abnormal levels can disrupt this balance. In obese girls, low adiponectin levels may contribute to earlier puberty as a reduced inhibition of *KISS1*/*GnRH* signalling can accelerate pubertal onset. In contrast, in obese boys, low adiponectin levels are associated with delayed puberty, likely due to its role in maintaining insulin sensitivity and testosterone production. In addition, excess adiposity increases oestrogen conversion (via aromatase activity), which may suppress testosterone and delay pubertal progression. Leptin, another key adipokine, can have significant effects. In obese girls, elevated leptin levels may promote the early activation of the *KISS1* system and precocious puberty. Leptin also stimulates CYP19A1 expression, enhancing oestrogen biosynthesis and leading to a higher oestrogen-to-testosterone ratio. In obese boys, this increase in oestrogen may suppress testosterone production and delay pubertal progression. Together, these findings underscore that adipokines, which are abundant in obese children, may modulate pubertal timing via both central (neuroendocrine) and peripheral (metabolic) pathways. Although a growing body of evidence supports these associations, the exact mechanisms remain unclear, necessitating further research. Further longitudinal studies are needed to investigate the effect that interventions such as weight loss would have on the duration of puberty. Importantly, early intervention to prevent or manage obesity in children could potentially mitigate the risk of pubertal disturbances and their long-term consequences.

## 8. Future Perspectives

The nuanced and multifactorial nature of pubertal development necessitates the need for further research in order to address the inconsistencies that exist in the current literature. Establishing a definitive causal relationship between body weight and puberty is a difficult task that faces multiple limitations. To begin, identifying the stage and onset of puberty can be difficult when taking body weight into account. This is especially true when staging breast development in girls as physicians must clinically be able to distinguish adipose tissue from breast tissue, which can lead to inconsistent results. Staging pubertal changes in boys can prove to be even more challenging as they lack determining markers such as menarche in girls. To elaborate, because clinicians use physiological changes that are secondary to the activation of the HPG axis, it would be beneficial to establish more accurate documentation in future literature, specifically by combining the documentation of these secondary sex characteristics with serum hormonal levels such as FSH, LH, and adipokines [74].

Additionally, rapidly shifting lifestyle patterns such as the excessive consumption of energy-dense foods and poor exercise habits [75] pose confounding factors affecting body weight that can skew results based on global regions and ethnicity [76,77,78,79]. As such, these factors must also be accounted for when discussing the effects of body weight and puberty. Longitudinal studies following children from childhood to adolescence could play a crucial role in understanding the nuanced involvement of lifestyle patterns on body weight and the effects on pubertal development.

Cohesively, obesity in children and adolescents is often determined using the BMI, which does not consider body fat distribution in adolescents as they reach their growth spurts. Therefore, more extensive measurements, such as the body adiposity index (BAI), could prove to be more beneficial in building a comprehensive hypothesis of the mechanistic pathways behind obesity and its effects on puberty [80].

Finally, detailed studies into adipokines such as resistin, visfatin, chemerin, and omentin should be taken into consideration in future literature when discussing obesity. The establishment of conclusive relationships between these adipokines and their effects on body weight could facilitate bridging the gap in understanding hormonal pathways, linking obesity to changes in pubertal timing [54].

## Figures and Tables

**Table 1:** Sexual dimorphism of the effects of Insulin and adipokines on the onset of puberty.

	Insulin	Adipokines (Leptin and Adiponectin)
Obesity in Boys	Delay in initiation of puberty secondary to the suppression of the HPG axis	Increased inhibition of the HPG axis that further contributes to the delay in the initiation of puberty
Obesity in Girls	Acceleration in initiation of puberty secondary to the positive feedback loop exerted on the HPG axis	Earlier activation of the HPG axis that further contributes to the acceleration of the initiation of puberty

## Data Availability

This is a review paper and does not include original data.

## Abbreviations

HPG	Hypothalamic–Pituitary–Gonadal
KISS1	Kisspeptin 1
MKRN3	Makorin Ring Finger Protein 3
EDCs	Endocrine-Disrupting Chemicals
GnRH	Gonadotropin-Releasing Hormone
BMI	Body Mass Index
FSH	Follicle Stimulating Hormone
LH	Luteinising Hormone
PA	Puberty-Activating
PI	Puberty-Inhibiting
NPY	Neuropeptide Y
LEP	Leptin
GABA	Gamma-Aminobutyric Acid
DLK1	Delta-Like Non-Canonical Notch Ligand 1
KISS1R	Kisspeptin Receptor
GPR54	G Protein-Coupled Receptor 54
DHEA	Dehydroepiandrosterone
DHEAS	Dehydroepiandrosterone Sulphate
HPA	Hypothalamic–Pituitary–Adrenal
WHO	World Health Organization
UNICEF	United Nations Children’s Fund
NCD-RisC	NCD Risk Factor Collaboration
DEHP	Di(2-ethylhexyl) phthalate
BBB	Blood–Brain Barrier
LEPR	Leptin Receptor
GH	Growth Hormone
CYP19A1	Cytochrome P450 19A1
AdipoR1	Adiponectin Receptor 1
AdipoR2	Adiponectin Receptor 2
IL-6	Interleukin-6
TNFα	Tumor Necrosis Factor-Alpha
SHBG	Sex Hormone-Binding Globulin
SGA	Small for Gestational Age
BAI	Body Adiposity Index
